# A method of determining anaerobic threshold from percutaneous oxygen saturation

**DOI:** 10.1038/s41598-022-24271-w

**Published:** 2022-11-22

**Authors:** Masatsugu Abe, Kai Ushio, Yuri Ishii, Yuki Nakashima, Daisuke Iwaki, Kouki Fukuhara, Makoto Takahashi, Yukio Mikami

**Affiliations:** 1 FANCL Corporation Research Institute, 2-13 Kamishinano, Totsuka-ku, Yokohama, Kanagawa 244-0806 Japan; 2 Department of Rehabilitation Medicine, Hiroshima University Hospital, 1-2-3, Kasumi, Minami-ku, Hiroshima, 734-8551 Japan; 3 Division of Rehabilitation, Department of Clinical Practice and Support, Hiroshima University Hospital, Hiroshima, Japan; 4 Department of Biomechanics, Graduate School of Biomedical and Health Sciences, Hiroshima University, Hiroshima, Japan

**Keywords:** Health care, Diagnosis, Disease prevention

## Abstract

The anaerobic threshold (AT) is the point of the aerobic-to-anaerobic metabolic switch. Despite the many clinical applications of AT, this measurement requires sophisticated equipment and skills. Here, we investigated a simple measurement method for AT using percutaneous oxygen saturation (SpO_2_) and pulse rate (PR) with a pulse oximeter in a study of exercise stress on healthy volunteers. Twenty individuals (ten men and ten women) were included in the study. Various respiratory parameters, including AT, were measured using conventional analytical methods. The SpO_2_ threshold (ST) was calculated using the SpO_2_-Slope method. The mean ± standard deviations SpO_2_ at ST was 97.8% ± 0.3% in men and 99.0 ± 0.3% in women. The concordance and interchangeability between ST and various five different types of AT, the ventilatory equivalent for oxygen (VE/VO_2__AT), V-Slope (V-Slope_AT), ventilatory equivalent (VE_AT), respiratory exchange ratio (R_AT), and partial pressure of end-tidal oxygen (PETO_2__AT) were generally high, with positive correlation coefficients in the range of [0.68–0.80]. These findings suggest that the SpO_2_-Slope method with a pulse oximeter may be a useful and simple method to determine AT compared to conventional methods.

## Introduction

Regular physical activity at an appropriate level is necessary to maintain health^1–4^. Previous studies have shown that individuals with a higher level of physical endurance have a healthier life, extended longevity, and lower mortality and that exercise improves cardiac function^5–9^. The anaerobic threshold (AT) is defined as the point at which the metabolism switches from aerobic to anaerobic. Exercising at the AT could prevent cardiovascular diseases and metabolic syndrome and contribute to the maintenance and improvement of health^10–13^. Exercising at AT is also used in sports training as it can help improve the athletes’ competitiveness^14^.

In the exercise above AT, glycolysis is accelerated, resulting in lactate accumulation and acidosis. Lactate is buffered by bicarbonate to produce CO_2_. This results in respiratory gas changes, such as increased alveolar CO_2_ emissions relative to oxygen uptake^15^. Consequently, the best-known methods of AT measurement are expiratory gas analysis and blood lactic acid quantification^16–19^. Expiratory gas analysis is a widely used noninvasive method in medicine and sports. However, it requires an expensive expiratory gas analyzer and a trained technician^20^. This method can determine five different types of AT, the ventilatory equivalent for oxygen (VE/VO_2__AT), V-Slope (V-Slope_AT), ventilatory equivalent (VE_AT), respiratory exchange ratio (R_AT), and partial pressure of end-tidal oxygen (PETO_2__AT), of which the VE/VO_2_-AT and V-Slope-AT are the most widely used^21,22^. It has been reported that during exercise loading, VE/VO_2_, R, and PETO_2_ movements flex upward at AT, the slope of the VCO_2_–VO_2_ relationship increases rapidly, and VE increases excretion^17,21,22^. As new, simple, accurate, and noninvasive methods of AT measurement are desirable, methods using the partial pressure of carbon dioxide (PaCO_2_), lactic acid in sweat, systolic blood pressure, and heart rate (HR) have been developed. However, none of them has been able to provide a practical alternative to expiratory gas analysis^23–25^.

Pulse oximetry is widely used in clinical practice as it provides a noninvasive method of measuring percutaneous oxygen saturation (SpO_2_), which reflects arterial oxygen pressure (PaO_2_)^26,27^. SpO_2_ during exercise is used in both clinical and research settings for many purposes, including the evaluation of the severity of cardiac functional impairment, the confirmation of the efficacy of exercise therapy, the assessment of the need for oxygen supplementation, and the evaluation of the oxygenation level of hemoglobin in arterial blood^28^. SpO_2_ is generally measured with pulse oximetry in the fingers, earlobes, or forehead. Notably, its accuracy is greatly affected by peripheral hypoperfusion and body movements, which hamper pulse wave detection^29,30^.

Previous studies have found that SpO_2_ declines during incremental exercise^26,30–32^. In athletes, the value of the inflexion point at which SpO_2_ suddenly declines during exercise is reportedly associated with AT^33^. During incremental exercise, there are two inflection points at which the SpO_2_ declines before AT. The time to the second decrease in SpO_2_ and the time before AT is reached are closely correlated^34^. However, SpO_2_ fluctuations during exercise are inconsistent and complex^34^. Visual determination of the inflexion point at which SpO_2_ suddenly starts to decline requires significant experience and knowledge of SpO_2_ from the investigator. Hence, a method to automatically determine AT using SpO_2_ and pulse rate (PR) with only a pulse oximeter, a noninvasive and simple device that does not require expiratory gas analysis or even HR monitor, would greatly contribute to improving the health and performance of more people from optimal intensity exercise using AT.

In this study, we developed a method to calculate AT automatically from SpO_2_ and PR data obtained with pulse oximetry and investigated its validity as an alternative method for AT measurement.

## Results

**Figure 1:**
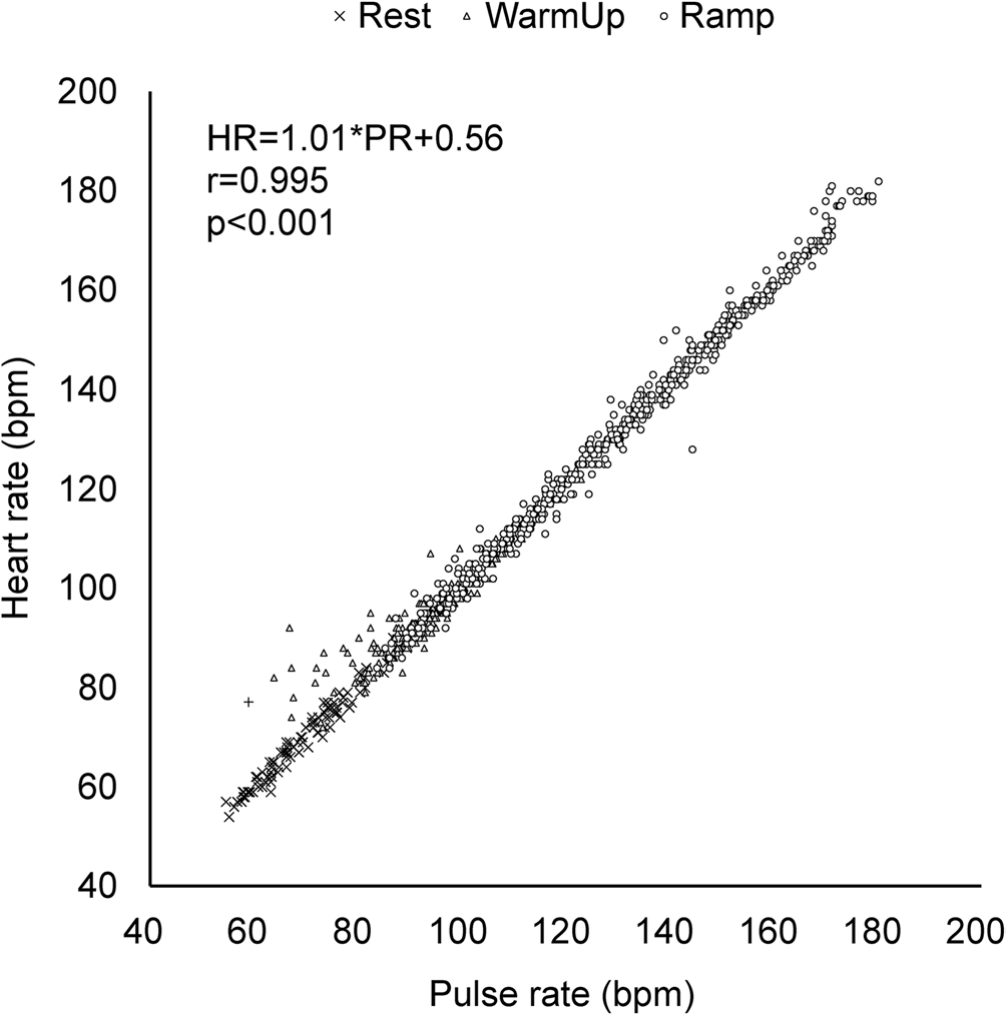
Relationship between pulse rate and heart rate. The data is from the rest to the end of loading. The pulse rate was measured with a pulse oximeter, and the heart rate was measured with a heart rate monitor. The correlation was investigated by calculating Pearson’s correlation coefficient (r) and the p-value. *Bpm* beats per minute, *HR* heart rate, *PR* pulse rate, *SpO*_*2*_ percutaneous oxygen saturation.

**Figure 2:**
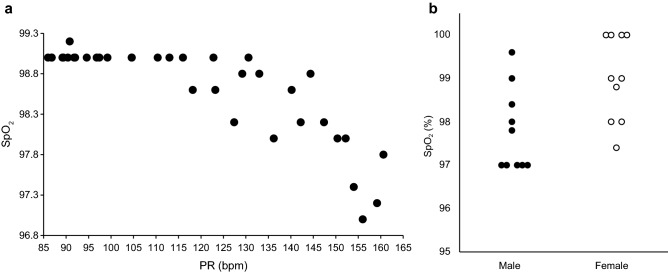
(**a**) Representative SpO_2_ and PR time series data during cardiopulmonary exercise testing. *SpO*_*2*_ percutaneous oxygen saturation, *PR* pulse rate, *Bpm* beats per minute. (**b**) Distribution of SpO_2_ corresponding to ST. SpO_2_ was distributed in the range of 97–100%. *SpO*_*2*_ percutaneous oxygen saturation, *ST* percutaneous oxygen saturation threshold.

The demographic data of the subjects are shown in Table 1. The mean age of the participants was 37.0 ± 2.1 years (25–57 years). Their mean body mass index (BMI) was 21.2 ± 0.4 kg/m^2^ (17.7–25.5 kg/m^2^). Figure 1 shows a scatter plot of the PR and HR of all subjects. There was a strong correlation between PR and HR from rest through cardiopulmonary exercise testing (CPX) (*r* = 0.995, *p* < 0.001). SpO_2_ and PR during cardiopulmonary exercise testing decrease at some point with increasing exercise intensity. Figure 2a shows representative SpO_2_ and PR time series data during cardiopulmonary exercise testing. SpO_2_ decreased from a certain point with increasing PR, although the behavior was not constant. Figure 2b shows SpO_2_ values at the SpO_2_ threshold (ST). In all subjects, SpO_2_ at ST appeared within the SpO_2_ reference range (96%-100%).

**Table 1:** Characteristics of the participants.

Parameter	Unit	Values	Min	Max
Number	n	20 (Male 10, Female 10)	–	–
Age	years	37.9 ± 2.1	25	57
Height	cm	163.6 ± 1.7	152.7	178.3
Weight	kg	56.9 ± 1.9	46.3	74.6
BMI	kg/m^2^	21.2 ± 0.4	17.8	25.5

Values are presented as mean ± SE.

*BMI* body mass index, *SE* standard error.

Table 2 shows the PR, oxygen consumption (VO_2_), and Load at different AT values and ST. For VE/VO_2__AT, R_AT, and PETO_2__AT, indeterminate AT data were treated as missing data. The final analysis included 19 subjects for VE/VO_2__AT and R_AT and 14 subjects for PETO_2__AR. The mean PR was 132.2 ± 3.1 at V-Slope_AT, 133.0 ± 2.8 at VE/VO_2__AT, 129.5 ± 3.1 at R_AT, 135.4 ± 4.0 at PETO_2__AT, 129.7 ± 3.3 at VE_AT, and 127.8 ± 2.9 at ST. The VO_2_ was 1408.6 ± 64.3 at V-Slope_AT, 1427.4 ± 74.4 at VE/VO_2__AT, 1364.8 ± 80.2 at R_AT, 1398.5 ± 79.4 at PETO_2__AT, 1366.4 ± 69.0 at VE_AT, and 1328.8 ± 44.6 at ST. The Load was 107.8 ± 4.8 at V-Slope_AT, 109.9 ± 5.5 at VE/VO_2__AT, 103.9 ± 6.0 at R_AT, 107.8 ± 5.6 at PETO_2__AT, 104.0 ± 5.0 at VE_AT, and 101.3 ± 2.7 at ST. Each individual's PR, oxygen consumption (VO_2_), and each AT and ST for Load are detailed in Supplementary Tables S1, S2, and S3.

**Table 2:** Pulse rate, VO_2,_ and Lad of each AT and ST.

Parameter (unit)	Breath analysis method					SpO_2_ method
	V-Slope_AT (n = 20)	VE/VO_2__AT (n = 19)	R_AT (n = 19)	PETO_2__AT (n = 14)	VE_AT (n = 20)	ST (n = 20)
Pulse rate (bpm)	132.2 ± 3.1	133.0 ± 2.8	129.5 ± 3.1	135.4 ± 4.0	129.7 ± 3.3	127.8 ± 2.9
VO_2_ (mL/min)	1408.6 ± 64.3	1427.4 ± 74.4	1364.8 ± 80.2	1398.5 ± 79.4	1366.4 ± 69.0	1328.8 ± 44.6
Load (watts)	107.8 ± 4.8	109.9 ± 5.5	103.9 ± 6.0	107.8 ± 5.6	104.0 ± 5.0	101.3 ± 2.7

Values are presented as mean ± SE.

*Bpm* beats per minute, *VO*_*2*_ oxygen consumption, *SpO*_*2*_ percutaneous oxygen saturation, *V-Slope* V-Slope, *VE/VO*_*2*_ oxygen ventilatory equivalent, *R* gas exchange ratio, *PETO*_*2*_ end-tidal oxygen concentration, *VE* ventilation, *AT* anaerobic threshold, *ST* percutaneous oxygen saturation threshold, *SE* standard error.

Table 3 shows the analysis of concordance, interchangeability, and correlation between ST and different AT values. The mean difference between PR at ST and that at R_AT and VE_AT (95% CI lower bound/upper bound) was − 1.5 (− 6.6/3.7) and − 1.9 (− 6.5/2.8), respectively, whereas the mean differences between PR at ST and that at V-Slope_AT, VE/VO_2__AT, and PETO_2__AT were − 4.3 (− 8.4/ − 0.3), − 5.3 (− 9.2/ − 1.4), and − 4.6 (− 9.2/ − 0.02), respectively. Addition errors were observed because the 95% CI did not include 0. The 20% relative error rate between ST and different AT values exceeded 95% in all cases, confirming the high interchangeability of the two sets of measurement results. The correlation coefficients were 0.68–0.80, indicating a positive correlation between the two sets of measurement results. The VO_2_ values also exhibited high interchangeability and a positive correlation between those at ST and those at different AT values. However, the mean differences with the values at V-Slope_AT, VE/VO_2__AT, and PETO_2__AT were − 79.8 (− 157.5/ − 2.2), − 111.5 (− 190.5/ − 32.6), and − 87.5 (− 168.5/ − 6.4), respectively. Addition errors were also observed for these findings. W values also showed high interchangeability and a positive correlation, with addition errors observed, between ST and VE/VO_2__AT and between ST and PETO_2__AT of 8.9 (− 15.8/ − 2.0) and − 7.2 (− 14.2/ − 0.2), respectively.

**Table 3:** Consistency, compatibility, and correlation of ST and AT.

Variables (n)	V-Slope_AT (20)	VE/VO_2__AT (19)	R_AT (19)	PETO_2__AT (14)	VE_AT (20)
**Pulse rate (bpm)**					
Average difference (d) (95% CI Lower/Upper)	− 4.3 (− 8.4/0.3)	− 5.3 (− 9.2/− 1.4)	− 1.5 (− 6.6/3.7)	− 4.6 (− 9.2/− 0.02)	− 1.9 (− 6.5/2.8)
± 20% relative error (%)	95	100	100	100	100
Correlation coefficient (r) (*p*-value)	0.79 (< 0.01)	0.80 (< 0.01)	0.68 (< 0.01)	0.84 (< 0.01)	0.75 (< 0.01)
**VO**_**2**_** (mL/min)**					
Average difference (d) (95% CI Lower/Upper)	− 79.8 (− 157.5/− 2.2)	− 111.5 (− 190.5/− 32.6)	− 40.6 (− 133.8/52.6)	− 87.5 (− 168.5/− 6.4)	− 37.7 (− 122.9/47.6)
± 20% relative error (%)	90	89	89	93	90
Correlation coefficient (r) (*p*-value)	0.83 (< 0.01)	0.92 (< 0.01)	0.89 (< 0.01)	0.91 (< 0.01)	0.83 (< 0.01)
**Load (watt)**					
Average difference (d) (95% CI Lower/Upper)	− 6.5 (− 13.0/0.1)	− 8.9 (− 15.8/− 2.0)	− 2.7 (− 10.9/5.5)	− 7.2 (− 14.2/− 0.2)	− 2.7 (− 10.0/4.6)
± 20% relative error (%)	90	89	74	93	80
Correlation coefficient (r) (*p*-value)	0.80 (< 0.01)	0.89 (< 0.01)	0.84 (< 0.01)	0.86 (< 0.01)	0.74 (< 0.01)

*Bpm* beats per minute, *VO*_*2*_ oxygen consumption, *V-Slope* V-Slope, *VE/VO*_*2*_ oxygen ventilatory equivalent, *R* gas exchange ratio, *PETO*_*2*_ end-tidal oxygen concentration, *VE* ventilation, *CI* confidence interval, *r* correlation coefficient, *AT* anaerobic threshold, *ST* percutaneous oxygen saturation threshold.

## Discussion

In this study, we investigated whether the automatic calculation of AT at ST using pulse oximetry was a valid alternative to the conventional method of AT measurement with expiratory gas analysis. Our results confirmed the validity of the SpO_2_-Slope method for determining AT from the two parameters of SpO_2_ and PR with a high degree of accuracy. The superiority of the SpO_2_-Slope method lies in its ability to automatically calculate ST as the inflexion point from the two parameters of SpO_2_ and PR. Since this approach does not require any special skills, experience, or knowledge, it offers a simpler method of AT measurement than conventional expiratory gas analysis.

It has been well established from arterial blood gas measurements that arterial oxygen partial pressure decreases during incremental exercise^35,36^. SpO_2_ measured with pulse oximetry reflects arterial blood oxygen saturation, which is associated with arterial oxygen partial pressure. In recent years, SpO_2_ has also been reported to decrease during incremental exercise^37^. In light of this observation, Nikooie et al. reported that the inflexion point at which SpO_2_ rapidly decreases occurs at the same load as AT^33^. Martín-Escudero et al*.* reported that there are two inflexion points at which SpO_2_ decreases before AT. Additionally, the time until the second decrease in SpO_2_ is strongly correlated with the time taken to reach AT^34^. The time until the second decrease in SpO_2_ is also moderately correlated with VO_2max_^34^_._ Therefore, it may even be possible to estimate exercise tolerance from the decrease in SpO_2_.

Arterial oxygen partial pressure and SpO_2_ are known to decrease during incremental exercise under a moderate load and as the AT is approached. However, this phenomenon has a complex underlying mechanism of action^34–37^. Accurate determination of the inflexion point at which SpO_2_ starts to decrease may be difficult with a visual inspection. In this regard, Martín-Escudero et al*.*^34^ did not mention the association between the decrease in SpO_2_ and AT. Had the method described in this study been used, Martín-Escudero et al*.* and many other studies might have been able to estimate AT from the inflexion point in SpO_2_.

Nikooie et al. found a correlation between HR at the inflection point of a sudden drop in SpO_2_ and HR at Lactate-AT (lactate threshold corresponding to a blood lactate level of 4 mmol/L)^33^. In our study, based on the premise that PR measured by pulse oximetry is highly correlated with HR measured by HR sensor, five representatives ATs, VE/VO_2__AT, V-Slope_AT, VE_AT, R_AT, and PETO_2__AT, measured by expiratory gas analysis, showed high correlation and agreement with SpO_2_-Slope method to obtain AT from the two parameters, SpO_2_, and PR. Therefore, our method to calculate AT automatically from SpO_2_ and PR data obtained by pulse oximetry can potentially be a noninvasive and simple alternative method of measuring AT. For SpO_2_ and PR measurements, a new medical wrist-worn device has been shown to be sufficiently accurate, reliable, and consistent compared to medical pulse oximeters, with no side effects^38^. In the future, our method could be extended to wearable devices to continuously measure SpO_2_ and PR in daily life, which could contribute to not only improving health and performance by providing optimal exercise intensity but also detecting cardiovascular diseases.

The SpO_2_ at ST, which reflects AT, was ≥ 96% in all our study subjects, with a mean value of 97.8% ± 0.3% in men and 99.0% ± 0.3% in women. All the subjects in the study of Martín-Escudero et al*.* were female athletes with a severe drop from the basal value of 98.07% ± 0.616 to 93.7% ± 1.65% before. This finding may correspond to exercise-induced arterial hypoxemia, defined as a drop in SpO_2_ of − 4% or more in comparison with the resting value^30,32^. According to Dominelli et al., many studies have identified a decrease in PaO_2_ corresponding to EIAH, even at submaximal exercise^39^. Other studies, however, have not identified the presence of EIAH in women at a load of 60% VO_2max_, at which AT may occur^39^. This finding suggests that the magnitude of the decrease in SpO_2_ may vary widely between individuals, affecting the analysis of our data.

The accuracy of SpO_2_ values measured with pulse oximetry also depends on the measurement devices^40^. Because SpO_2_ measurements during exercise are affected by body movements and hypoperfusion, this study used a medical device that has been verified to produce highly accurate results even when the body is moving, and perfusion is low^29^. In addition, discrepancies between HR and PR also occur in conditions of body movement and hypoperfusion^41^. An older study reported that HR deviates from PR when HR exceeds 155 beats/min (bpm)^45^. In our study, we confirmed that PR measured by pulse oximetry and HR measured by the reliable HR sensor^42^ showed a high correlation. This did not change even when HR exceeded 155 bpm (Fig. 1). As a result, there was no adverse effect of exercise, and the decrease in SpO_2_ was likely small. A fingertip sensor was used for SpO_2_ measurements both in our study and in other studies, in which SpO_2_ measured with pulse oximetry decreased before AT^33,34^. In light of factors such as body movement, the use of sensing at the ears or forehead, which are less affected by body movement, may enable more accurate measurement of AT from SpO_2_ and PR in the future.

This study had several limitations. First, as the subjects were healthy adult volunteers, the results may not be applicable to elderly people and patients with diseases requiring exercise rehabilitation or to highly trained competitive athletes. Second, the exercise in this study was performed on a bicycle ergometer. Therefore, it is not known whether similar results would be obtained from exercise on a treadmill or steps. Third, in this experiment, we measured SpO_2_ and PR at a fingertip. However, other sites may enable more sensitive SpO_2_ and PR measurements that are unaffected by body movements during exercise. Forth, the subjects who participated in this experiment were only Japanese. Fawzy et al. have reported higher rates of potential hypoxemia undetected by pulse oximetry in Asian, black, and Hispanic patients compared to non-Hispanic white patients^43,44^. Therefore, it is necessary to keep in mind that racial bias could exist in pulse oximetry measurements due to skin pigmentation. Fifth, in the CPX of our study, the mixing chamber method was used, which resulted in a longer interval between breath gas analysis and HR measurements. Further studies are required to verify whether these measurements are affected by (1) expanding the range of subjects to include elderly, sick patients, and competitive athletes, (2) the use of different types of exercise, (3) the measurements of SpO_2_ at different sites, (4) multiracial with different skin colors and (5) breath-by-breath method for short expiratory gas parameters and HR responses. In addition, this study has a convergent validity, and a test–retest would further ensure the reliability of this study.

In summary, this is the first study to show that the ST calculated using the SpO_2_-Slope method from SpO_2_ and PR results during exercise testing exhibits high concordance, interchangeability, and correlation with AT values measured with expiratory gas analysis. Our results suggested that the SpO_2_-Slope method may be a valid simple, inexpensive, and accurate method of AT measurement. The simplicity, low cost, and high accuracy of this technique of AT measurement decrease both the burden on the subject and the analysis cost. As such, this approach has the potential to make major contributions in areas including the prevention and improvement of metabolic syndrome, exercise therapy for respiratory and cardiac rehabilitation, and the extension of healthy longevity as an index for building endurance in improving sports competitiveness.

## Methods

### Study design

This study was approved in advance by the FANCL Corporation. Clinical Research Ethics Review Board (C2021-006, approval date of April 30, 2021) and conducted at the FANCL Corporation. Research Institute in May and June 2021. The experiments complied with the Declaration of Helsinki (adopted in 1964, revised for the 7th time at the Fortaleza General Meeting in 2013) and the Ethical Guidelines for Medical and Biological Research Involving Human Subjects (December 22, 2014, partly revised February 28, 2017). Care was always taken to protect the subjects’ human rights. The study participants were provided with a full written explanation of the study purpose and its content, as well as the voluntary nature of their participation. Participants gave both their informed consent for study participation and for the publication of their images/data in an online open-access publication. The UMIN clinical trial registration system number for this study was UMIN000044183. It was registered with UMIN on 12/05/2021 and last updated on 17/11/2021 (https://center6.umin.ac.jp/cgi-open-bin/ctr/ctr.cgi?function=brows&action=brows&recptno=R000050457&type=summary&language=J).

### Study subjects

This clinical trial included ten men and ten women. The selection criteria of the study were as follows: healthy men and women aged between 20 and 65 years with a BMI of 18.5–30 kg/m^2^. The exclusion criteria were as follows: (1) serious liver, gastrointestinal, kidney, or heart disease; (2) participation in a long-term interventional study of food or medicinal product either at enrollment or during the study (including planned participation); (3) intention to become pregnant during the study period, pregnancy (including possible pregnancy), or lactation; (4) COVID-19 infection or close contact with an infected person; (5) exercise-induced arrhythmia, exercise-induced anaphylaxis, or other exercise-induced condition or motor disorder; (6) considered unsuitable for study participation for any other reasons by a study investigator. After consent had been obtained, the subjects’ date of birth, age, current medical history (treatment, medication), exercise habits, smoking, and alcohol consumption were analyzed.

### Experimental methods

The subjects were instructed to make no major changes to their normal diet, exercise, sleep cycle, or other aspects of their daily lives during the three days preceding the experiment; to avoid vigorous exercise, alcohol consumption, and binge eating and drinking during the day preceding the experiment; and to consume their evening meals by 10 p.m. on the evening before the experiment with avoidance of excessively fatty food. The experiment was conducted in the morning after the subjects had fasted (with the exception of fluid intake) since the previous evening. On the day of the test, the subjects consumed a designated food 3 h before and subsequently ate and drank nothing but water until the test was conducted. The designated food was a Lime and Grapefruit Flavor Calorie Mate Gel (Otsuka Pharmaceutical, Japan) with a nutritional content of 200 kcal, including 8.2 g protein, 4.4 g fat, 33.2 g carbohydrate, and salt equivalence of 0.11 g. Before the test, their height, weight, body fat percentage, blood pressure, and pulse rate were measured.

### Study parameters

**Figure 3:**
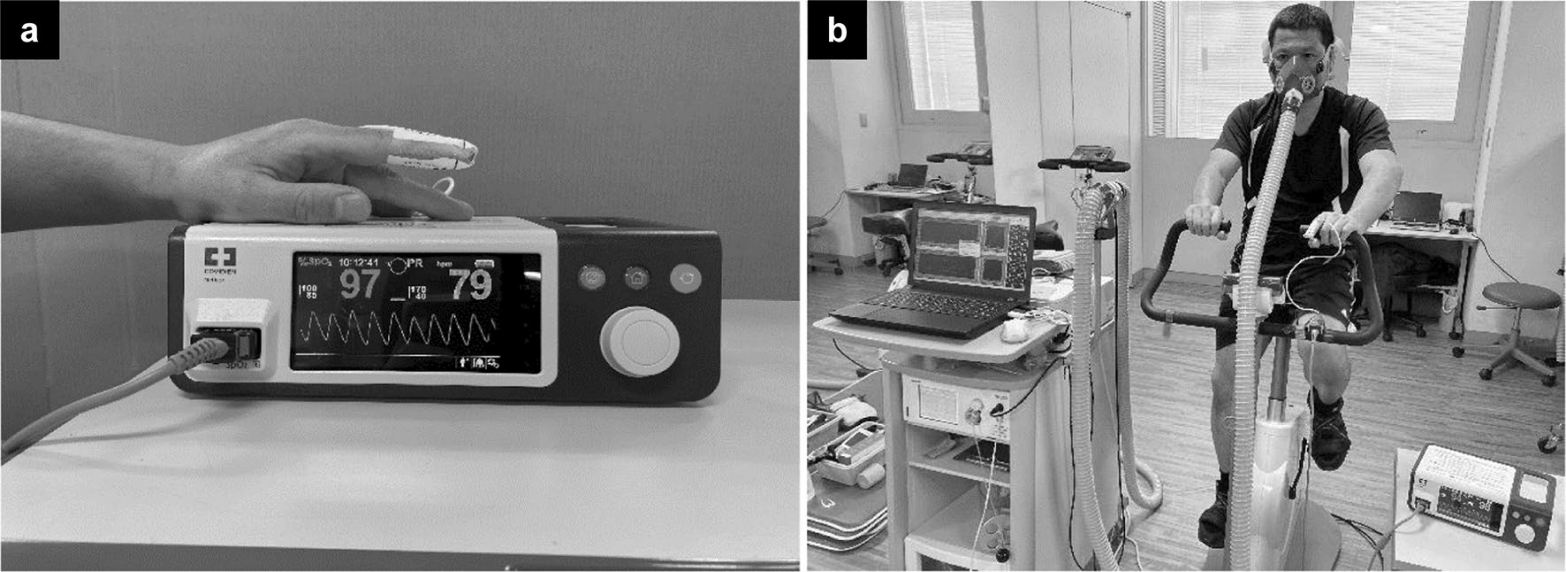
(**a**) SpO_2_ measurement device and sensor attachment site. SpO_2_ and pulse rate were measured with pulse oximetry via a sensor attached to the index finger of the left hand. *SpO*_*2*_ percutaneous oxygen saturation. (**b**) Cardiopulmonary exercise testing using the ergometer and pulse oximeter. *Participants gave both their informed consent for study participation and for the publication of their images/data in an online open-access publication.

An online expiratory gas analyzer (AE300S, Minato Medical Science, Tokyo, Japan) was used to measure VO_2_, carbon dioxide emission (VCO_2_), ventilation (VE), gas exchange ratio (R = VCO_2_/VO_2_), oxygen ventilatory equivalent (VE/VO_2_), carbon dioxide ventilatory equivalent (VE/VCO_2_), end-tidal oxygen concentration (PETO_2_), and end-tidal carbon dioxide concentration (PETCO_2_) in expiratory gases sampled using the mixing chamber method. HR was measured with a heart rate sensor (T31 Heart Rate Sensor N, POLAR, Japan) attached to the chest. Simultaneously, SpO_2_ and pulse rate (PR) were measured with pulse oximetry (NellcorTM N-BSJ, Covidien Japan) via a sensor (Nelcor Sensor DS100A, Covidien Japan) attached to the index finger of the left hand as Fig. 3a. Expiratory gas parameters and HR were measured every 20 s, while SpO_2_ and PR were measured every 4 s, with the mean values of every five measurements (20-s mean values) used as the measured values. Data from the final minute of the warm-up until the end of the test were used in the analysis.

### Cardiopulmonary exercise testing method

Cardiopulmonary exercise testing (CPX) was conducted by means of a Ramp Test using an ergometer (Corival cpet, Kyokko Bussan, Japan) as Fig. 3b (Participants gave both their informed consent for study participation and for the publication of their images/data in an online open access publication.). After 2 min at rest, the subject engaged in a 5-min warm-up at 50 W and 120 rpm, after which the exercise load was increased by 10 W/min until the subject reached one of the conditions for halting the test. These conditions include any of the followings: (1) the subject was unable to continue exercise at 120 rpm due to leg fatigue; (2) the subject’s heart rate during the exercise exceeded 85% of the predicted maximum heart rate (220 − age in years), or (3) the investigator decided that the test should be halted. The saddle was adjusted so that the subject’s knees were slightly bent when the pedals were at their lowest point.

### Determination of AT

AT determination with expiratory gas analysis was conducted by doctors and physiotherapists involved in the CPX. Following the criteria adopted by Wasserman et al*.*, five different ATs were measured: V-Slope_AT (defined as the point at which the VO_2_–VCO_2_ relationship with increasing exercise intensity increased to ≥ 45°); VE/VO_2__AT (defined as the point at which VE/VO_2_ started to increase with no increase in VE/VCO_2_); R_AT (defined as the point at which R started to increase); PETO_2__AT (defined as the point at which PETO_2_ started to increase with no increase in PETCO_2_); and VE_AT (defined as the point at which VCO_2_ started to increase in proportion to VO_2_)^17,21,22^. Graphs were produced using VO_2_ as the independent variable for V-Slope_AT and PR as the independent variable for the other methods. The PR and load corresponding to VO_2_ of V-Slope_AT were calculated with linear regression. For the other four methods, the VO_2_ and load corresponding to the PR of AT were calculated with linear regression.

### Automatic determination of AT using SpO_2_ and PR with a pulse oximeter

**Figure 4:**
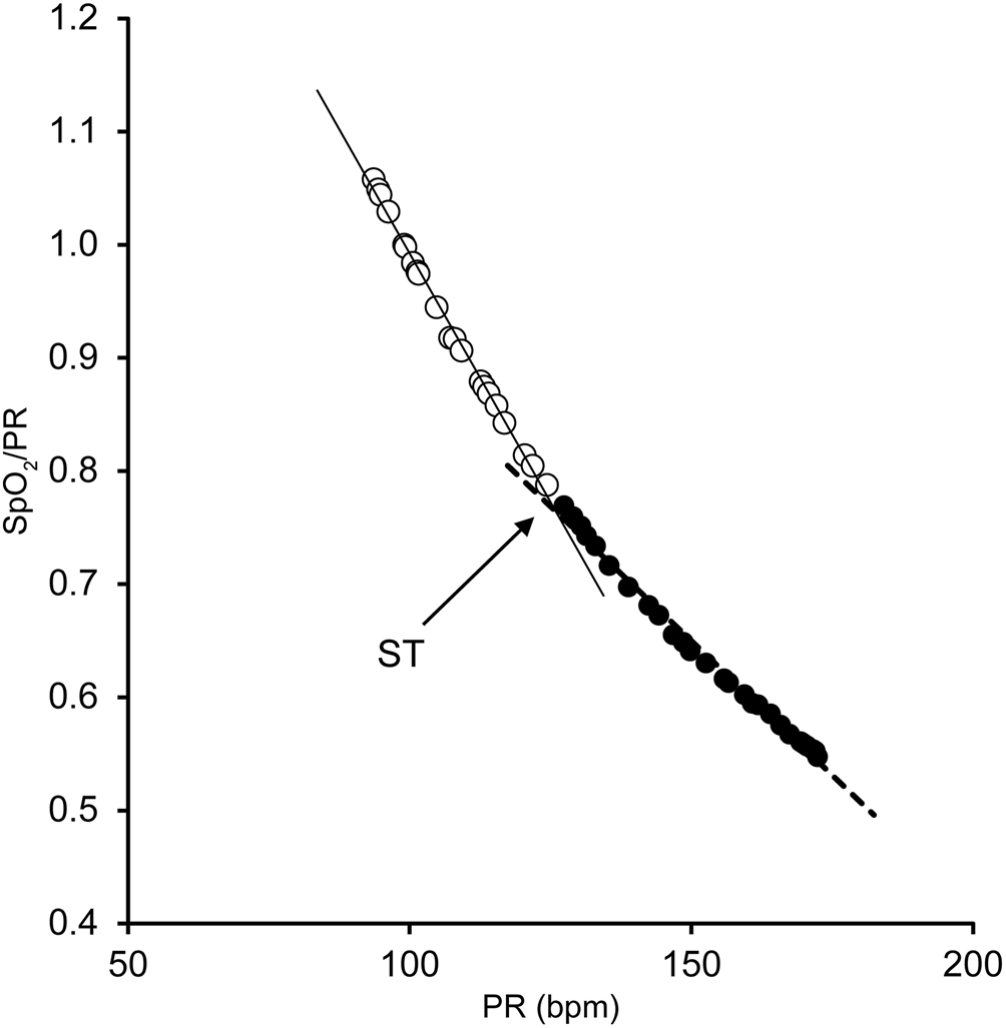
SpO_2__Slope method for creating ST. ST is the dividing point where the sum of squared residuals of the two regression lines (SpO_2_/PR and PR) is minimized. *SpO*_*2*_ percutaneous oxygen saturation, *ST* percutaneous oxygen saturation threshold, *PR* pulse rate, *Bpm* beats per minute.

SpO_2_ and PR during the CPX were measured using a pulse oximeter. Figure 4 shows the SpO_2__Slope method for creating the SpO_2_ threshold (ST). We calculated the dividing point that minimized the residual sum of squares when the pulse oximetry measurements were divided into two regression lines at a bifurcation on the 2nd-order regression curve. This regression curve was obtained when PR was designated as the independent variable and SpO_2_/PR as the dependent variable. Of the two points between this dividing point, the one with the higher PR was chosen as ST. The VO_2_ and load corresponding to PR at ST were calculated with linear regression. SpO_2_ of PR at ST was designated as ST_SpO_2_.

### Statistical analysis

We used the following tests to investigate the concordance, interchangeability, and correlation between the exercise intensity (PR, VO_2_, load) at AT, determined using the five different expiratory gas analysis methods, and at ST, determined with the SpO_2_-Slope method. Concordance was investigated by calculating the 95% confidence interval (95% CI) of the mean difference between the two measured values (d). If this CI included 0, then the concordance was considered high. If it did not include 0, an addition error was considered to be present. Interchangeability was investigated by calculating the difference between the mean measurement (a) and measurement (b) for each subject. The error was calculated as a proportion of the difference to the mean (relative error) (b/a × 100). If the proportion of data for which the relative error was ± 20% (20% relative error rate) exceeded 75%, the two methods were considered interchangeable. The correlation was investigated by calculating Pearson’s correlation coefficient (*r*) and the *p-*value. If 0.4 < |*r*| ≤ 0.7, the parameters were considered correlated. If 0.7 < |*r*| ≤ 1.0, they were considered strongly correlated. JMP^®^14.1.0 statistical software (SAS Institute Inc.) was used for statistical analysis.

## Supplementary Information

Supplementary Tables.

## Data Availability

All data from these studies are contained within this manuscript or are available from the corresponding author upon reasonable request. Source data are provided in this paper.
