# Supplementary material for: A method of determining anaerobic threshold from percutaneous oxygen saturation
Source: Sci Rep. 2022 Nov 22;12:20081. doi: 10.1038/s41598-022-24271-w (PMC9684533; doi:10.1038/s41598-022-24271-w)
Supplement: Supplementary file 1 — Supplementary Tables. [file 41598_2022_24271_MOESM1_ESM.docx]

**A method of determining anaerobic threshold from percutaneous oxygen saturation**

Masatsugu Abe^1^, Kai Ushio^2*^, Yuri Ishii^1^, Yuki Nakashima^3^, Daisuke Iwaki^3^, Kouki Fukuhara^3^, Makoto Takahashi^4^, Yukio Mikami^2^

^1^ FANCL Corporation Research Institute, 2-13 Kamishinano, Totsuka-ku, Yokohama, Kanagawa Prefecture 244-0806, Japan. Tel: +81-45-820-3628

^2^ Department of Rehabilitation Medicine, Hiroshima University Hospital, Hiroshima, Japan. 1-2-3, Kasumi, Minami-ku, Hiroshima, 734-8551, Japan. Tel: +81-82-257-5566

^3^ Department of Clinical Practice and Support, Hiroshima University Hospital, Hiroshima, Japan

^4^ Department of Biomechanics, Graduate School of Biomedical and Health Sciences, Hiroshima University, Hiroshima, Japan

**Corresponding author:** *

Kai Ushio

ORCiD: 000-0001-5195-1808

1-2-3, Kasumi, Minami-ku, Hiroshima, 734-8551, Japan

Telephone number: +81-82-257-5566

Fax number: +81-82-257-5594

E-mail: [ushiosista@gmail.com](mailto:ushiosista@gmail.com)

**Acknowledgements**

We would like to thank all the participants, everyone involved in this study and in the writing of this paper.

**Author contributions**

Conceptualization, methodology and exercise tests were conducted by M.A. Data analysis was performed by K.U., Y.N., D.I., K.F., M.T. and Y.M. Y.I performed the statistical analysis and created the figures. The original draft was prepared by M.A. and Y.I. Review and editing were performed by M.A. K.U., Y.N., D.I., K.F., M.T., Y.M., and K.U. supervised the entire process. All authors have read and agreed to the published version of the manuscript.

**Funding**

This study was funded by a research grant from FANCL Corporation.

**Competing interests**

The authors declare no competing interests.

**Supplementary Table S1.** Pulse rate of AT and ST

| **Subject ID** | **Pulse rate (bpm)** | | | | | |
| --- | --- | --- | --- | --- | --- | --- |
|  | **Breath analysis method** | | | | | **SpO_2_ method** |
|  | **V-Slope_AT （n = 20）** | **VE/VO_2__AT （n = 19）** | **R_AT （n = 19）** | **PETO_2__AT （n = 14）** | **VE_AT （n = 20）** | **ST （n = 20）** |
| 1 | 136 | 132 | 129 | 132 | 130 | 127 |
| 2 | 157 | 156 | 150 | 170 | 160 | 162 |
| 3 | 150 | 151 | 143 | 153 | 153 | 140 |
| 4 | 137 | 143 | 129 | － | 117 | 140 |
| 5 | 127 | 120 | 110 | 110 | 110 | 124 |
| 6 | 122 | 122 | 122 | － | 122 | 119 |
| 7 | 125 | 134 | － | 133 | 133 | 123 |
| 8 | 123 | 124 | 117 | 121 | 131 | 128 |
| 9 | 149 | 139 | 139 | 135 | 139 | 127 |
| 10 | 148 | 150 | 150 | 150 | 150 | 147 |
| 11 | 110 | 125 | 121 | － | 106 | 106 |
| 12 | 113 | 125 | 119 | 124 | 125 | 116 |
| 13 | 141 | 141 | 136 | 142 | 136 | 138 |
| 14 | 141 | 141 | 141 | 136 | 139 | 124 |
| 15 | 142 | 136 | 131 | 131 | 133 | 116 |
| 16 | 131 | 128 | 128 | 132 | 128 | 128 |
| 17 | 118 | 109 | 103 | － | 102 | 114 |
| 18 | 128 | 126 | 123 | 126 | 125 | 128 |
| 19 | 134 | ­－ | 151 | － | 134 | 130 |
| 20 | 111 | 125 | 119 | ­－ | 121 | 117 |
| Mean | 132.2 | 133.0 | 129.5 | 135.4 | 129.7 | 127.8 |
| SE | 3.1 | 2.8 | 3.1 | 4.0 | 3.3 | 2.9 |

**Supplementary Table S2.** VO2 of AT and ST

| **Subject ID** | **VO_2_ (mL/min)** | | | | | |
| --- | --- | --- | --- | --- | --- | --- |
|  | **Breath analysis method** | | | | | **SpO_2_ method** |
|  | **V-Slope_AT （n = 20）** | **VE/VO_2__AT （n = 19）** | **R_AT （n = 19）** | **PETO_2__AT （n = 14）** | **VE_AT （n = 20）** | **ST （n = 20）** |
| 1 | 1288 | 1224 | 1176 | 1224 | 1192 | 1139 |
| 2 | 1121 | 1106 | 1016 | 1316 | 1166 | 1202 |
| 3 | 1625 | 1644 | 1494 | 1681 | 1681 | 1446 |
| 4 | 1294 | 1404 | 1148 | － | 929 | 1353 |
| 5 | 1282 | 1155 | 973 | 973 | 973 | 1235 |
| 6 | 1152 | 1152 | 1152 | － | 1152 | 1116 |
| 7 | 1446 | 1591 | － | 1575 | 1575 | 1417 |
| 8 | 789 | 799 | 731 | 770 | 866 | 839 |
| 9 | 1890 | 1669 | 1669 | 1581 | 1669 | 1413 |
| 10 | 1047 | 1072 | 1072 | 1072 | 1072 | 1030 |
| 11 | 1612 | 2001 | 1897 | － | 1508 | 1508 |
| 12 | 1318 | 1610 | 1464 | 1585 | 1610 | 1401 |
| 13 | 1467 | 1467 | 1388 | 1483 | 1388 | 1420 |
| 14 | 1917 | 1917 | 1917 | 1821 | 1879 | 1600 |
| 15 | 1815 | 1713 | 1628 | 1628 | 1662 | 1373 |
| 16 | 1477 | 1422 | 1422 | 1495 | 1422 | 1418 |
| 17 | 1183 | 1044 | 951 | － | 935 | 1118 |
| 18 | 1413 | 1375 | 1319 | 1375 | 1357 | 1420 |
| 19 | 1638 | － | 1911 | － | 1638 | 1575 |
| 20 | 1399 | 1760 | 1605 | － | 1657 | 1553 |
| Mean | 1408.6 | 1427.4 | 1364.8 | 1398.5 | 1366.4 | 1328.8 |
| SE | 64.3 | 74.4 | 80.2 | 79.4 | 69.0 | 44.6 |

**Supplementary Table S3.** Load of AT and ST

| **Subject ID** | **Load (watts)** | | | | | |
| --- | --- | --- | --- | --- | --- | --- |
|  | **Breath analysis method** | | | | | **SpO_2_ method** |
|  | **V-Slope_AT （n = 20）** | **VE/VO_2__AT （n = 19）** | **R_AT （n = 19）** | **PETO_2__AT （n = 14）** | **VE_AT （n = 20）** | **ST （n = 20）** |
| 1 | 104 | 98 | 93 | 98 | 95 | 90 |
| 2 | 84 | 83 | 74 | 104 | 89 | 92 |
| 3 | 127 | 128 | 115 | 131 | 131 | 111 |
| 4 | 100 | 110 | 88 | － | 69 | 105 |
| 5 | 102 | 91 | 75 | 75 | 75 | 98 |
| 6 | 86 | 86 | 86 | － | 86 | 88 |
| 7 | 106 | 118 | － | 117 | 117 | 104 |
| 8 | 68 | 69 | 60 | 65 | 78 | 75 |
| 9 | 157 | 139 | 139 | 131 | 139 | 117 |
| 10 | 85 | 88 | 88 | 88 | 88 | 84 |
| 11 | 124 | 159 | 149 | － | 115 | 115 |
| 12 | 95 | 117 | 106 | 116 | 117 | 102 |
| 13 | 115 | 115 | 108 | 116 | 108 | 111 |
| 14 | 141 | 141 | 141 | 133 | 138 | 115 |
| 15 | 138 | 130 | 123 | 123 | 125 | 102 |
| 16 | 110 | 105 | 105 | 112 | 105 | 104 |
| 17 | 93 | 80 | 71 | － | 70 | 87 |
| 18 | 103 | 100 | 95 | 100 | 98 | 104 |
| 19 | 114 | － | 137 | － | 114 | 108 |
| 20 | 104 | 132 | 120 | － | 124 | 116 |
| Mean | 107.8 | 109.9 | 103.9 | 107.8 | 104.0 | 101.3 |
| SE | 4.8 | 5.5 | 6.0 | 5.6 | 5.0 | 2.7 |

ID: identification; Bpm: beats per minute; VO_2_: oxygen consumption; SpO_2_: percutaneous oxygen saturation; V-Slope: V-Slope; VE/VO_2_: oxygen ventilatory equivalent; R: gas exchange ratio; PETO_2_: end-tidal oxygen concentration; VE: ventilation; AT: anaerobic threshold; ST: percutaneous oxygen saturation threshold; SE: standard error.

Rate. Bpm: beats per minute.
